# Differences in Heart Rate Variability and Baroreflex Sensitivity between Male and Female Athletes

**DOI:** 10.3390/jcm12123916

**Published:** 2023-06-08

**Authors:** M. Abdullah Shafiq, Cody A. Ellingson, Gregory P. Krätzig, Kim D. Dorsch, J. Patrick Neary, Jyotpal Singh

**Affiliations:** 1 Faculty of Kinesiology and Health Studies, University of Regina, 3737 Wascana Pkwy, Regina, SK S4S 0A2, Canada; muhammad.a.shafiq25@gmail.com (M.A.S.); cellingson9@outlook.com (C.A.E.); kim.dorsch@uregina.ca (K.D.D.); patrick.neary@uregina.ca (J.P.N.); 2 Department of Psychology, University of Regina, 3737 Wascana Parkway, Regina, SK S4S 0A2, Canada; greg.kratzig@uregina.ca

**Keywords:** heartbeat, cardiovascular system, autonomic function, controlled respiration, sex differences, blood pressure

## Abstract

Heart rate variability (HRV), systolic blood pressure variability (BPV), and spontaneous baroreflex sensitivity (BRS) are indirect and approximate measures of autonomic regulation of the cardiovascular system. Studies have shown differences in HRV and BRS between males and females; however, no study has observed differences in BPV, HRV, or BRS between male and female athletes. One hundred males (age 21.2 ± 2.1 y; BMI 27.4 ± 4.5 kg/m^2^) and sixty-five females (age: 19.7 ± 1.6 y; BMI 22.7 ± 2.2 kg/m^2^) were assessed during the pre-season baseline. We collected resting beat-to-beat blood pressure and R-R intervals using finger photoplethysmography and a 3-lead electrocardiogram, respectively. Participants underwent a controlled slow breathing protocol (six breaths/minute: 5 s inhale, 5 s exhale) for 5 min. Spectral and linear analysis was conducted on blood pressure and ECG data. Regression curves were fitted to the blood pressure and R-R signals, with the slopes providing the BRS parameters. Male athletes had significantly (*p* < 0.05) lower mean heart rate, RR interval SD2/SD1, HRV % low-frequency, and higher BP high-frequency power during controlled respiration. No differences were found in any BRS parameters. HRV and BPV responses to a slow breathing protocol differed between male and female athletes; however, BRS responses did not.

## 1. Introduction

Heart rate variability (HRV), systolic blood pressure variability (BPV), and spontaneous baroreflex sensitivity (BRS) are markers for assessing autonomic and vascular function [1,2,3,4]. Heart rate variability refers to the variation in time intervals between consecutive heartbeats due to heart–brain interactions and autonomic nervous system (ANS) processes [5]. Low HRV potentially indicates increased stress [6] and inflammation [7,8], and can be determined using time-domain or frequency-domain analysis [5]. Time-domain analysis describes the variability of interbeat interval between successive heartbeats, whereas frequency-domain analysis provides an estimated distribution of absolute or relative power in frequency bands [5]. 

The baroreflex helps regulate arterial blood pressure (BP) [9] under varying conditions [10]. In response to blood pressure changes, the baroreflex shifts sympathetic tone through a negative feedback loop by adjusting HR. Baroreflex sensitivity (BRS) is the change in the interbeat interval when blood pressure changes [11] and is measured by regression of the R-R interval and systolic blood pressure [12].

Blood pressure variability (BPV) represents the fluctuations in beat-to-beat blood pressure [13]. BPV provides information about the autonomic regulation of the cardiovascular system and is thought to be influenced by central and reflex autonomic modulations, which are regulated by arterial and cardiopulmonary reflexes [13]. 

There is a lack of research that differentiates HRV, BRS, and BPV between males and females while controlling respiration. Respiratory rates cause fluctuations in heart rate, blood pressure, and R-R intervals [3,14,15], consequently influencing HRV, BRS, and BPV values [11,15]. Given these findings, controlling respiratory rate between participants is important when making comparisons. This study aimed to address this gap and determine if there are differences in HRV, BRS, and BPV between male and female athletes when controlling respiration. Given that cardiac differences exist between males and females during rest [16], we hypothesized that differences would exist between males and females in various HRV, BRS, and BPV parameters.

## 2. Materials and Methods

### 2.1. Participants

We conducted baseline physiological testing [17] on all varsity-level athletes at the University of Regina. Athletes from basketball, football, hockey, volleyball, and soccer were recruited from June 2022 to October 2022, and all assessments took place between 10 a.m. and 5 p.m. Volunteer participants signed a consent form and were informed to refrain from caffeine for 6 h, strenuous exercise for 12 h, and alcohol for 24 h before the testing [11]. 

### 2.2. Data Collection

The assessment started with five minutes of seated rest to determine baseline physiology, followed by five minutes of six-breaths-per-minute (0.1 Hz) seated controlled breathing. A breathing frequency of 0.1 Hz helps to prevent complexities associated with respiratory sinus arrhythmia [17] and elicits the respiratory-induced baroreflex, influencing blood pressure and heart rate [11,15]. The six-breaths-per-minute technique has been previously described and is considered to be an appropriate technique for measuring indices of cardiovascular autonomic function while controlling respiratory rate [11,15,17]. Furthermore, the European Society of Hypertension has recently stated that the usefulness of the analysis of continuous beat-to-beat recordings is questionable without ensuring that respiration frequency is taken into consideration [18].

We collected beat-to-beat BP using finger photoplethysmography, with the height correction unit placed on the participant’s heart level and the finger cuff placed around the left middle finger (NOVA and Finometer, Finapres Medical Systems BV, Enschede, The Netherlands). We recorded R-R interval data using a three-lead electrocardiogram (ECG) (Lead I configuration). We sampled the raw data signals at 1000 Hz and used PowerLab and LabChart software to obtain a visual representation (AD Instruments, Colorado Springs, CO, USA).

### 2.3. Data Analysis

First, the BP and ECG waveforms were visually inspected for anomalies. We included participants with a valid baseline assessment, ensuring a lack of artifacts in BP and ECG waveform [11,17]. We then analyzed the R-R intervals from the ECG signals for assessment of HRV using spectral analysis, resulting in low-frequency (LF), high-frequency (HF), total power (TP), and LF/HF ratio values. The relative power was expressed as percent (%) HF and LF. Intervals derived from Poincaré plots provided short-term variability (SD1) and long-term variability (SD2). Furthermore, we combined beat-to-beat systolic BP and R-R intervals and analyzed BRS and BPV using the Ensemble-R software (Elucimed Ltd., Auckland, NZ, USA). 

Ensemble-R (Elucimed Ltd., Auckland, NZ, USA) provided systematic and monotonic three-beat increases or decreases in systolic BP and R-R intervals during controlled respiration [19]. The values were then paired as xy-coordinates to create a regression curve fitted to the BP and R-R signals [20]. We averaged slopes with correlation values of at least 0.8 for 300 s, which provided the upward sequences (BRS-up), downward sequences (BRS-down), and the average of the protocol (BRS-pooled) [21]. Spectral analysis was conducted to assess BPV. HF and LF power were measured at 0.15–0.40 Hz and 0.04–0.15 Hz, respectively, for HRV and BPV. 

### 2.4. Statistical Analysis

All data are presented as Mean ± SD. Normality was assessed using the Shapiro–Wilk test in R Studio (RStudio Inc., Boston, MA, USA), followed by either an unpaired t-test or a Wilcoxon rank sum test depending on normality. Additionally, an exploratory multiple regression analysis was conducted to determine if age, BMI, type of sport played, and history of concussion [22] contributed to the significant outcomes. Statistical significance for all assessments was set at *p* < 0.05. 

## 3. Results

Out of the 225 participants who completed the study, 100 males (age 21.2 ± 2.1 y; BMI 27.4 ± 4.5 kg/m^2^) and 65 females (age: 19.7 ± 1.6 y; BMI 22.7 ± 2.2 kg/m^2^) met our inclusion criteria. See Table 1 for participant demographics.

### 3.1. Five Min Seated Rest

Males had a significantly lower HR mean (mean = 70.1 bpm, *p* = 0.018) and higher RR mean (mean = 0.88 s, *p* = 0.044) compared to females [HR mean (mean = 74.4 bpm, *p* = 0.018); RR mean (mean = 0.84 s, *p* = 0.044); Table 2].

### 3.2. Five Min Controlled Respiration

Males had significantly lower HR mean (mean = 73.4 bpm, *p* = 0.014), SD2/SD1 (mean = 3.1, *p* = 0.013), and LF% (mean = 84.0, *p* = 0.004) compared to females [HR mean (mean = 77.4 bpm, *p* = 0.014); SD2/SD1 (mean = 3.4, *p* = 0.013); LF% (mean = 86.8, *p* = 0.004)]. For BPV, males had a significantly higher BP HF power (mean = 5.6, *p* = 0.043) compared to females [BP HF power (mean = 4.5, *p* = 0.043); Table 2]. 

### 3.3. Multiple Regression Analysis

The analysis used HR Mean, RR Mean, LF%, SD2/SD1, BP HF Power, BRS- up, BRS- down, and BRS Pooled as the dependent variables. Significance was found for the HR Mean [F(5, 145) = 2.57, *p* = 0.029, R^2^ = 0.081], RR Mean [F(5, 145) = 2.44, *p* = 0.037, R^2^ = 0.078], LF% [F(5, 145) = 2.31, *p* = 0.047, R^2^ = 0.074], BP HF Power [F(5, 145) = 3.11, *p* = 0.011, R^2^ = 0.097], and BRS-up [F(5, 145) = 3.52, *p* = 0.0089, R^2^ = 0.089] models. BMI added significance to the prediction for LF% (*p* = 0.02) and BP HF Power (*p* = 0.002). The type of sport played added significance to BRS-up (*p* = 0.002), BRS-down (*p* = 0.03), and BRS-pooled (*p* = 0.003) models. 

## 4. Discussion

We assessed HRV, BPV, and BRS differences between male and female athletes to add comparative values to the literature and to observe differences between the two groups. Specifically, we found that when utilizing the same respiratory rate at 0.1 Hz, males have a higher RR Mean and HF-BPV, while females have a higher HR Mean, %LF-HRV, and SD2/SD1. These findings suggest that differences exist between male and female athletes and should be considered when doing any baseline physiological assessments. Consequently, this study provides important information to further understand cardiovascular parameters among athletes and between sexes. Under resting conditions, males tend to have a greater respiratory rate as compared to females [23]. In turn, the differences in respiratory rate can influence cardiovascular function, including heart rate and stroke volume. Using a controlled breathing protocol at six breaths per minute, we were able to ensure that differences in breathing frequencies were not related to the observed outcomes. As shown in the results, the differences in HRV and BPV were more obvious during controlled respiration as compared to spontaneous breathing. This highlights the importance of controlling respiration when assessing these parameters, as shown in previous studies [11,15,17].

A recent study has shown differences in the cardiac cycle timing intervals between male and female athletes [16], and many studies have displayed differences in HRV, BPV, and BRS between sexes [24,25,26,27]. Our results coincide with many studies illustrating significant differences in cardiovascular parameters between sexes [24,25,26,27]. 

In many age groups, there are significant differences in HRV between males and females [24]. Females tend to have higher SDNN and RMSSD values [24], although not during controlled respiration. A meta-analysis also showed females having a greater HR mean and a smaller RR mean [26], which corresponds with our own findings.

Regarding BRS, Fu and Ogoh [28] found no significant differences in sympathetic BRS between males and females. However, they noted that BRS decreased faster in aging females than males. Similarly, our findings showed no significant difference in BRS pooled, BRS-up, and BRS-down between male and female athletes during controlled respiration. Pertaining to BPV, research shows that males tend to have higher BPV compared to females when assessing 24 h BPV [25]; however, there is a lack of literature determining beat-to-beat BPV spectral analysis between healthy males and females. Nevertheless, the literature does show that males tend to have higher BPV compared to females when assessing clinical populations or after physiological stress [29,30]. We also found that males had a higher HF-BPV power compared to females during similar respiration rates. This suggests that there is a greater parasympathetic dominance influencing the BPV from the breathing rate for male athletes as compared to females. 

Much of the research discussing HRV, BRS, and BPV differences mainly examine the differences in clinical populations with an underlying condition or individuals over 25 [24,29,30,31,32,33]. The cardiovascular changes may be attributed to anthropometric differences between athletes, although more research is required to elucidate these findings. Our preliminary, exploratory regression models found that the combination of BMI, age, type of sport, and history of concussion can be potential contributors to the cardiovascular metrics, with BMI being a particularly strong predictor of LF% and BP HF Power, and the type of sport being a strong predictor of BRS parameters. In other research, age has been shown to influence many cardiovascular parameters [24,27,34], while BMI has an inverse relationship with HRV [35] and BRS [36], and a direct relationship with BPV [37].

Our study presents limitations that will be addressed with future research. First, there are unequal sample sizes in the two groups, as well as a wide distribution of different sports that the athletes play. Second, we only had 5 min of resting data, thus limiting our insights into HF and LF parameters. Furthermore, we only utilized a six-breaths-per-minute assessment. While the six-breaths-per-minute protocol is considered to be a valid approach to assess cardiovascular autonomic parameters [11,15,17], this does represent a deviation from the expected spontaneous respiratory rate, and can thus result in an external stressor to the athletes. Utilizing multiple breathing frequencies can provide more insights to the influence of respiration on HRV, BRS, and BPV. Finally, we did not control for variables such as female menstrual cycle and time of day. These variables may have limited our data and should be controlled in future studies.

## 5. Conclusions

This is the first study looking at HRV, BPV, and BRS differences between sexes while controlling respiration. The results showed significant differences in mean heart rate, mean R-R intervals, SD2/SD1, % low-frequency, and BP high-frequency power values between varsity-level male and female athletes. We assessed these cardiovascular parameters during seated rest and controlled respiration. There were significant differences in HRV during both rest and controlled breathing, and in BPV during the controlled breathing protocol only. Therefore, we showed differences in cardiovascular parameters between males and females, and provide comparative values of HRV, BRS, and BPV to the literature. Future research should include details pertaining to the respiratory cycle and saturation levels to understand how the chemoreceptor reflex may influence these findings.

## Figures and Tables

**Table 1:** Participant demographics.

Parameters	Males (*n* = 100) Mean ± SD	Females (*n* = 65) Mean ± SD	*p*-Value
Age (y) *	21.2 ± 2.1	19.7 ± 1.6	<0.001
Height (m) *	1.84 ± 0.07	1.70 ± 0.07	<0.001
Body Mass (kg) *	93.3 ± 16.8	66.3 ± 9.2	<0.001
BMI (kg/m^2^) *	27.4 ± 4.5	22.7 ± 2.2	<0.001

* *p* < 0.05.

**Table 2:** Cardiovascular parameters during seated rest and controlled respiration.

Parameters	Males (*n* = 100) Mean ± SD	Females (*n* = 65) Mean ± SD	*p*-Value
HRV During Seated Rest			
HR Mean (bpm) *	70.1 ± 9.2	74.4 ± 12.2	0.018
RR Mean (ms) *	880 ± 120	840 ± 140	0.044
SDNN (ms)	66 ± 25	66 ± 30	0.98
LF%	53.91 ± 17.26	52.94 ± 18.48	0.74
HF%	28.67 ± 16.29	31.77 ± 18.26	0.27
LF/HF	2.99 ± 2.50	3.10 ± 3.69	0.83
SD1 (ms)	36.0 ± 21.6	34.7 ± 22.6	0.72
SD2 (ms)	85.8 ± 30.1	86.0 ± 37.7	0.97
SD2/SD1	2.73 ± 0.79	2.89 ± 0.95	0.28
HRV During Controlled Respiration			
HR Mean (bpm) *	73.4 ± 8.5	77.4 ± 10.1	0.014
RR Mean (ms) *	850 ± 100	810 ± 120	0.044
SDNN (ms)	0.12 ± 0.03	0.11 ± 0.03	0.22
LF% *	84.0 ± 7.08	86.8 ± 5.4	0.004
HF%	10.08 ± 5.79	8.69 ± 5.04	0.106
LF/HF	12.5 ± 10.4	13.6 ± 8.0	0.055
SD1 (ms)	54.4 ± 26.6	47.43 ± 22.81	0.074
SD2 (ms)	155.1 ± 45.7	143.39 ± 44.84	0.28
SD2/SD1 *	3.1 ± 0.64	3.4 ± 0.66	0.013
BPV During Seated Rest			
SBP	114.53 ± 10.71	114.50 ± 13.65	0.99
BP HF Power (mmHg^2^)	7.64 ± 6.5	6.99 ± 3.93	0.43
BP LF Power (mmHg^2^)	22.77 ± 15.7	23.38 ± 20.7	0.84
BP Total Power (mmHg^2^)	60.5 ± 35.6	55.2 ± 32.7	0.33
BPV During Controlled Respiration			
SBP	112.85 ± 11.95	112.32 ± 14.13	0.84
BP HF Power (mmHg^2^) *	5.6 ± 3.6	4.5 ± 2.9	0.043
BP LF Power (mmHg^2^)	76.69 ± 45.2	73.37 ± 39.5	0.62
BP Total Power (mmHg^2^)	109.0 ± 51.2	100.4 ± 44.2	0.25
BRS During Seated Rest			
BRS Pooled (ms/mmHg)	16.9 ±10.3	16.7 ± 9.1	0.88
BRS–up (ms/mmHg)	17.8 ± 12.1	17.0 ± 11.6	0.68
BRS–down (ms/mmHg)	16.0 ± 8.9	16.5 ± 9.1	0.75
BRS During Controlled Respiration			
BRS Pooled (ms/mmHg)	19.2 ± 9.5	18.9 ± 9.0	0.85
BRS–up (ms/mmHg)	24.1 ± 12.85	23.4 ± 12.7	0.70
BRS–down (ms/mmHg)	15.41 ± 8.1	15.5 ± 7.8	0.92

* *p* < 0.05. HR = Heart rate; RR = RR interval; SDNN: standard deviation of RR intervals; LF% = Percent Low-Frequency; HF% = Percent High-Frequency; LF = Low-Frequency; HF = High-Frequency; SD2 = long-term variability; SD1 = short-term variability; BP = Blood Pressure; SBP = Systolic Blood Pressure; HRV = Heart Rate Variability; BPV = Blood Pressure Variability; BRS = spontaneous Baroreflex Sensitivity.

## Data Availability

The datasets are available upon request to the corresponding author.

## References

[1] Ellingson C.J., Singh J., Ellingson C.A., Dech R., Piskorski J., Neary J.P. (2022). The Influence of External Stressors on Physiological Testing: Implication for Return-to-Play Protocols. Curr. Res. Physiol..

[2] (1996). Heart Rate Variability: Standards of Measurement, Physiological Interpretation and Clinical Use. Task Force of the European Society of Cardiology and the North American Society of Pacing and Electrophysiology. Circulation.

[3] Parati G., Saul J.P., di Rienzo M., Mancia G. (1995). Spectral Analysis of Blood Pressure and Heart Rate Variability in Evaluating Cardiovascular Regulation. Hypertension.

[4] Pinna G.D., Maestri R., la Rovere M.T. (2015). Assessment of Baroreflex Sensitivity from Spontaneous Oscillations of Blood Pressure and Heart Rate: Proven Clinical Value?. Physiol. Meas.

[5] Shaffer F., Ginsberg J.P. (2017). An Overview of Heart Rate Variability Metrics and Norms. Front. Public Health.

[6] Kim H.-G., Cheon E.-J., Bai D.-S., Lee Y.H., Koo B.-H. (2018). Stress and Heart Rate Variability: A Meta-Analysis and Review of the Literature. Psychiatry Investig..

[7] McCraty R., Shaffer F. (2015). Heart Rate Variability: New Perspectives on Physiological Mechanisms, Assessment of Self-Regulatory Capacity, and Health Risk. Glob. Adv. Health Med..

[8] Cooper T.M., McKinley P.S., Seeman T.E., Choo T.-H., Lee S., Sloan R.P. (2015). Heart Rate Variability Predicts Levels of Inflammatory Markers: Evidence for the Vagal Anti-Inflammatory Pathway. Brain Behav. Immun..

[9] Taylor C.E., Willie C.K., Ainslie P.N., Tzeng Y.-C. (2014). Assessment of Human Baroreflex Function Using Carotid Ultrasonography: What Have We Learnt?. Acta Physiol..

[10] Heusser K., Tank J., Luft F.C., Jordan J. (2005). Baroreflex Failure. Hypertension.

[11] Ellingson C.J., Singh J., Ellingson C.A., Sirant L.W., Krätzig G.P., Dorsch K.D., Piskorski J., Neary J.P. (2022). Alterations in Baroreflex Sensitivity and Blood Pressure Variability Following Sport-Related Concussion. Life.

[12] Filgueiras-Rama D. (2018). Sympathetic Innervation and Cardiac Arrhythmias. Cardiac Electrophysiology: From Cell to Bedside.

[13] Parati G., Ochoa J.E., Lombardi C., Bilo G. (2013). Assessment and Management of Blood-Pressure Variability. Nat. Rev. Cardiol..

[14] Russo M.A., Santarelli D.M., O’Rourke D. (2017). The Physiological Effects of Slow Breathing in the Healthy Human. Breathe.

[15] Guzik P., Piskorski J., Krauze T., Schneider R., Wesseling K.H., Wykretowicz A., Wysocki H. (2007). Correlations between the Poincaré Plot and Conventional Heart Rate Variability Parameters Assessed during Paced Breathing. J. Physiol. Sci..

[16] Singh J., Ellingson C.J., Ellingson C.A., Scott P., Neary J.P. (2023). Cardiac Cycle Timing Intervals in University Varsity Athletes. Eur. J. Sport Sci..

[17] Neary J., Singh J., Bishop S., Dech R., Butz M., Len T. (2019). An Evidence-Based Objective Study Protocol for Evaluating Cardiovascular and Cerebrovascular Indices Following Concussion: The Neary Protocol. Methods Protoc..

[18] Parati G., Bilo G., Kollias A., Pengo M., Ochoa J.E., Castiglioni P., Stergiou G.S., Mancia G., Asayama K., Asmar R. (2023). Blood Pressure Variability: Methodological Aspects, Clinical Relevance and Practical Indications for Management—A European Society of Hypertension Position Paper ∗. J. Hypertens.

[19] Parati G., Di Rienzo M., Bertinieri G., Pomidossi G., Casadei R., Groppelli A., Pedotti A., Zanchetti A., Mancia G. (1988). Evaluation of the Baroreceptor-Heart Rate Reflex by 24-Hour Intra-Arterial Blood Pressure Monitoring in Humans. Hypertension.

[20] McMillan N.J., Soares R.N., Harper J.L., Shariffi B., Moreno-Cabañas A., Curry T.B., Manrique-Acevedo C., Padilla J., Limberg J.K. (2022). Role of the Arterial Baroreflex in the Sympathetic Response to Hyperinsulinemia in Adult Humans. Am. J. Physiol.-Endocrinol. Metab..

[21] Tzeng Y.C., Sin P.Y.W., Lucas S.J.E., Ainslie P.N. (2009). Respiratory Modulation of Cardiovagal Baroreflex Sensitivity. J. Appl. Physiol..

[22] Bishop S.A., Dech R.T., Guzik P., Neary J.P. (2018). Heart Rate Variability and Implication for Sport Concussion. Clin. Physiol. Funct. Imaging.

[23] LoMauro A., Aliverti A. (2021). Sex and Gender in Respiratory Physiology. Eur. Respir. Rev..

[24] Voss A., Schroeder R., Heitmann A., Peters A., Perz S. (2015). Short-Term Heart Rate Variability—Influence of Gender and Age in Healthy Subjects. PLoS ONE.

[25] Thayer J.F., Sollers J.J., Friedman B.H., Koenig J. (2016). Gender Differences in the Relationship between Resting Heart Rate Variability and 24-Hour Blood Pressure Variability. Blood Press.

[26] Koenig J., Thayer J.F. (2016). Sex Differences in Healthy Human Heart Rate Variability: A Meta-Analysis. Neurosci. Biobehav. Rev..

[27] Laitinen T., Hartikainen J., Vanninen E., Niskanen L., Geelen G., Länsimies E. (1998). Age and Gender Dependency of Baroreflex Sensitivity in Healthy Subjects. J. Appl. Physiol..

[28] Fu Q., Ogoh S. (2019). Sex Differences in Baroreflex Function in Health and Disease. J. Physiol. Sci..

[29] Kappus R.M., Ranadive S.M., Yan H., Lane-Cordova A.D., Cook M.D., Sun P., Harvey I.S., Wilund K.R., Woods J.A., Fernhall B. (2015). Sex Differences in Autonomic Function Following Maximal Exercise. Biol. Sex Differ.

[30] Theodorakopoulou M., Karagiannidis A., Alexandrou M.-E., Pella E., Karpetas A., Baksiova A., Tsouchnikas I., Papagianni A., Sarafidis P. (2022). Sex Differences in Ambulatory Blood Pressure Trajectories and Blood Pressure Variability in Hemodialysis Patients. J. Hypertens..

[31] del Pinto R., Pietropaoli D., Dobre M., Ferri C. (2020). Prognostic Importance of Long-Term SBP Variability in High-Risk Hypertension. J. Hypertens..

[32] Williams D.P., Joseph N., Gerardo G.M., Hill L.K., Koenig J., Thayer J.F. (2022). Gender Differences in Cardiac Chronotropic Control: Implications for Heart Rate Variability Research. Appl. Psychophysiol. Biofeedback.

[33] Lan Y., Liu H., Liu J., Zhao H., Wang H. (2019). Gender Difference of the Relationship between Arterial Stiffness and Blood Pressure Variability in Participants in Prehypertension. Int. J. Hypertens..

[34] Laitinen T., Hartikainen J., Niskanen L., Geelen G., Länsimies E. (1999). Sympathovagal Balance Is Major Determinant of Short-Term Blood Pressure Variability in Healthy Subjects. Am. J. Physiol.-Heart Circ. Physiol..

[35] Speer K.E., Koenig J., Telford R.M., Olive L.S., Mara J.K., Semple S., Naumovski N., Telford R.D., McKune A.J. (2021). Relationship between Heart Rate Variability and Body Mass Index: A Cross-Sectional Study of Preschool Children. Prev. Med. Rep..

[36] Konstantinidou S., Argyrakopoulou G., Tentolouris N., Karalis V., Kokkinos A. (2021). Interplay between Baroreflex Sensitivity, Obesity and Related Cardiometabolic Risk Factors (Review). Exp. Ther. Med..

[37] Chen H., Zhang R., Zheng Q., Yan X., Wu S., Chen Y. (2018). Impact of Body Mass Index on Long-Term Blood Pressure Variability: A Cross-Sectional Study in a Cohort of Chinese Adults. BMC Public Health.

